# Phonetically Grounded Structural Bias in Learning Tonal Alternations

**DOI:** 10.3389/fpsyg.2021.705766

**Published:** 2021-07-26

**Authors:** Tingyu Huang, Youngah Do

**Affiliations:** Department of Linguistics, The University of Hong Kong, Hong Kong, China

**Keywords:** learning bias, simplicity, phonetic naturalness, artificial grammar learning, tone alternation

## Abstract

This study investigates the hypothesis that tone alternation directionality becomes a basis of structural bias for tone alternation learning, where “structural bias” refers to a tendency to prefer uni-directional tone deletions to bi-directional ones. Two experiments were conducted. In the first, Mandarin speakers learned three artificial languages, with *bi-directional* tone deletions, *uni-directional, left-dominant* deletions, and *uni-directional, right-dominant* deletions, respectively. The results showed a learning bias toward *uni-directional, right-dominant* patterns. As Mandarin tone sandhi is right-dominant while Cantonese tone change is lexically restricted and does not have directionality asymmetry, a follow-up experiment trained Cantonese speakers either on left- or right-dominant deletions to see whether the right-dominant preference was due to L1 transfer from Mandarin. The results of the experiment also showed a learning bias toward right-dominant patterns. We argue that structural simplicity affects tone deletion learning but the simplicity should be grounded on phonetics factors, such as syllables’ contour-tone bearing ability. The experimental results are consistent with the findings of a survey on other types of tone alternation’s directionality, i.e., tone sandhi across 17 Chinese varieties. This suggests that the directionality asymmetry found across different tone alternations reflects a phonetically grounded structural learning bias.

## Introduction

Experimental work has focused on two types of learning biases in phonological pattern learning: structural bias, a bias that favors patterns involving simple featural specifications, and substantive bias, a bias that favors phonetically natural patterns (Moreton and Pater, 2012a,b). In terms of structural bias, experimental work using artificial language learning paradigms shows that patterns involving more phonological features are harder to learn than patterns involving fewer phonological features (Moreton and Pater, 2012a). Previous work defined structural bias in phonology with distinctive features involved in phonological classifications or processes. A wide variety of phonological-learning experiments support the structural bias hypothesis, showing that a pattern with a single feature is easier to learn than ones with more features (Saffran and Thiessen, 2003; Peperkamp et al., 2006; Cristià and Seidl, 2008; Kuo, 2009; Skoruppa et al., 2009; Chambers et al., 2010). For example, a pattern distinguished as [p t k] vs. [b d g] is easier to acquire than a pattern [p d k] vs. [b t g] in an artificial language learning experiment (Saffran and Thiessen, 2003), because the former pattern only depends on a voicing contrast and thus is easier to learn, while the latter involves two featural contrasts manner of articulation (voiced vs. voiceless) and place of articulation (alveolar vs. labial vs. velar). When the number of relevant features is controlled, learning is better facilitated by dependencies between phonological components involving a single feature than by dependencies between those involving two different features (Moreton and Pater, 2012a). For instance, in learning stimuli of the shape C_1_V_1_C_2_V_2_, participants performed better with patterns of height agreement between the two vowels or voice agreement between the two consonants than with patterns showing a correlation between the height of a vowel and voicing of a consonant (Moreton, 2012). In addition to the learning of phonemes and phonotactics, work on morphophonological learning also supports the structural bias hypothesis (Pycha et al., 2003; Finley, 2008; Moreton, 2008; Finley and Badecker, 2009; Ettlinger et al., 2014). While a few exceptions exist (e.g., Finley and Badecker, 2010), most of the existing experimental research provides supporting evidence for the structural bias hypothesis (see Moreton and Pater, 2012a for an overview).

The primary focus of previous work on structural bias in phonology, however, has been on segments. As far as we know, only a few artificial language learning studies examined the role of biases in learning phonological patterns defined by suprasegmental features (see section “Biases in Learning Suprasegmental Features”), and their focus has been mainly on the role of “substantive bias” (Wilson, 2006). The substantive bias hypothesis predicts that patterns grounded on phonetic factors are better learned than those that are not. Accordingly, research on substantive bias in learning suprasegmental features has investigated whether suprasegmental patterns that are phonetically better-grounded are more readily learnable than those that are not (e.g., Carpenter, 2010; Zhang and Lai, 2010; Kao, 2017) (see section “Biases in Learning Suprasegmental Features”).

This study aims to fill a gap in the literature by testing the structural bias hypothesis in learning suprasegmental features. Our focus is on tones. Like segments, tones exhibit a variety of alternations cross linguistically, such as tone sandhi, tone spreading, and tone deletion (Gussenhoven, 2004). Also like segments, tones’ alternation patterns are distributed asymmetrically; for example, among Chinese varieties showing local substitution sandhi patterns, right-dominant patterns are more prevalent than left-dominant ones (Zhang, 2007). If the asymmetries in segmental phonology can be attributed, at least partially, to a structural bias effect in learning, we believe that the same should be tested for the learning of suprasegmental features. We hypothesize that structural bias affects the learning of tone alternation patterns, which in turn will shape the distributions of tone alternations in natural language. In this paper our specific focus is to understand whether the directionality asymmetry (i.e., bidirectional vs. unidirectional) found among tone alternation patterns in Chinese is reflected in learning.

The directionality of tone alternations is characterized by the position that remains unchanged after the application of an alternation (Chen, 2000; Zhang, 2007). An example of uni-directional, right-dominant pattern is Tianjin tone sandhi: 11^1^ + 11 → 24 + 11; 24 + 24 → 55 + 24 (Zhang and Liu, 2016), where the right tone remains unchanged and the left tone undergoes change. An example of uni-directional, left-dominant pattern is Chengdu tone sandhi: 45 → 44/T^2^ __; 13 → 11/T__ (Lin, 2015), where the right tone undergoes alternation, and the left tone remains unchanged. Chaoyang provides an example of bi-directional tone sandhi: 53 → 31/T __; 55 → 11/__T (Lee, 2002), where either the left or the right tone alternates.

Based on a survey conducted by Huang and Do (2021), where they examined the directionality of tone alternations in Chinese varieties and showed that uni-directional patterns are more prevalent than bi-directional ones, we predict that learners of tone alternations will prefer uni-directional patterns, other things being equal. We explore the role of such structural bias in learning tone alternations in two tone language populations, one with attested tone alternations conditioned by adjacent tones (Mandarin) and one without such tone alternation (Cantonese). The type of tone alternation tested in this study is tone deletion, which is unattested in either Mandarin or Cantonese, to minimize the first language transfer effect.^3^ In Experiment 1, Mandarin native speakers are exposed to one of three artificial languages: a language with uni-directional, left-dominant patterns (Language LD), a language with uni-directional, right-dominant patterns (Language RD), and a language with bi-directional tone alternation patterns (Language BD). “Bi-directional” refers to two different patterns toward different directions, e.g., a half of right-dominant and another half of left-dominant. Each language exhibits alternation patterns showing a vowel fusion and a tone deletion (e.g., fu35 + an55 → fin55. See section “Stimuli” for the details). In disyllabic tone deletions, Language BD preserves the tone either on the left or right syllable (bi-directional alternation); Language LD consistently preserves the tone on the left syllable (uni-directional, left-dominant alternation); Language RD consistently preserves the tone on the right syllable (uni-directional, right-dominant alternation). If the learning of tone alternation patterns is biased toward structurally simpler patterns, we predict that the two languages with uni-directional alternations (Languages LD and RD) will be learned better than Language BD which exhibits bi-directional alternations.

## Background

This section first reviews work on learning biases in suprasegmental phonology. We show that little attention has been paid to structural bias in learning phonological patterns defined by suprasegmental features, while some work reported substantively biased learning of stress and tone patterns. We then define structural complexity of tone alternations’ directionality. We also summarize a survey on tone alternation patterns in Chinese varieties by Huang and Do (2021), which shows skewed distributions toward uni-directional patterns than bi-directional ones. This section ends with the background of tone alternations in Mandarin and in Cantonese, the two languages of the target populations of the current study.

### Biases in Learning Suprasegmental Features

Previous studies on learning biases in suprasegmental patterns have provided support for the substantive bias hypothesis (Wilson, 2006). For instance, Carpenter (2010, 2016) investigated the learning of stress patterns using artificial language learning paradigms. Carpenter (2010) explored the learning of phonetically natural vs. unnatural stress patterns by adult native speakers of English and French. In the natural condition, stress occurs on low vowels; in the unnatural condition, stress occurs on high vowels. Stress on low vowels is phonetically grounded because low vowels have greater duration and more acoustic energy than high vowels (Clements, 1990), which increases perceptual salience. The results showed that both English and French participants learned the natural patterns better than the unnatural patterns. Carpenter (2016) found that 9- and 10-year-old children also performed better on the natural pattern over the unnatural pattern in learning the same artificial stress patterns. Kao (2017) survey of 30 tonal languages of Africa showed that a low level tone is always deleted when two adjacent vowels at a word boundary undergo deletion. The tendency to preserve a high level tone over a low level tone is phonetically grounded, because a high level tone is perceptually more salient than a low level tone (Yip, 2002, cited in Kao, 2017). In Kao’s experiment, English and Mandarin Chinese native speakers were trained on either phonetically grounded or ungrounded tone deletion patterns where one of two adjacent vowels was deleted (V_1_ + V_2_ → V_1_ or V_2_); in the grounded patterns, a high tone was retained (H + L → H; L + H → H); in the unnatural patterns, a low tone was retained (H + L → L; L + H → L). The participants were then tested on the items they had seen during the training as well as on unseen items. The results showed that the English speakers generalized the high tone retention patterns better than the low tone retention patterns both among the seen and unseen items, supporting the substantive bias hypothesis. The Mandarin speakers performed equally well on the two patterns for the seen items, but those who learned the high tone retention patterns were more likely to generalize the learned patterns to unseen items, compared to those who had learned the low tone retention patterns. Taken together, these results support a learning bias toward phonetically grounded and typologically common tone alternation patterns. While previous studies have reported supporting evidence for the substantive bias in learning patterns defined by suprasegmental features, no study has investigated the role of structural bias in tone learning.

The nature of substantive bias found from the learning of suprasegmental phonology is similar to that of segmental phonology in that the learning of phonological patterns grounded on phonetic substances are better than their ungrounded counterparts. If substantive bias plays a role in learning suprasegmental features in phonology, it is plausible to test the structural bias, another type of learning bias widely attested in segmental phonology, in the learning of suprasegmental features. The following section explains how we define structural complexity specifically for the directionality of tone alternations.

### Defining Structural Complexity of Tone Alternations’ Directionality

First, in the literature on non-linguistic pattern learning, psychological experiments have revealed that the learning difficulty increases as the number of relevant *features* increases, feature being defined in non-linguistic units, such as color, shape, or size (Shepard et al., 1961; Neisser and Weene, 1962; Nosofsky et al., 1994; Feldman, 2000; Love, 2002; Smith et al., 2004). For instance, participants were shown geometric figures that vary in color (black vs. white), shape (circle vs. triangle), or size (large vs. small). In each cycle, they were shown a figure and were required to judge whether it belongs to the target concept. The results showed that the difficulty increased along with the number of relevant features, e.g., when the figures differed only in color vs. when they differed in both color and shape (Shepard et al., 1961). If we subscribe to the idea that linguistic and non-linguistic pattern learning are, to a certain degree, comparable (Moreton et al., 2017), the directionality of tone alternations can be considered a target “feature” of learning. If so, we predict that a uni-directional tone alternation will be easier to learn than a bi-directional tone alternation pattern, because the number of directions involved in the former system is one while the latter is two.

Second, a bi-directional system is more complicated than a uni-directional system, due to its *high uncertainty*: it is *uncertain* whether the tone on the right syllable or the left syllable will remain in a bi-directional system, thus can be viewed as a more complex system. In contrast, a uni-directional system provides consistent and absolute evidence to learners as to the direction of tone alternations. In fact, it is not unprecedented to incorporate predictability into the consideration of structural complexity. A complex system has proved difficult to learn, as shown from human brain research (Ladyman et al., 2013). Complexity theorists in social science argued that a complex system is intrinsically unpredictable (Prigogine and Stengers, 1984; Urry, 2003). If we were to incorporate the degree of predictability into our consideration of structural complexity, the predictions would be as follows: In a uni-directional system, the tone on a specific position always alternates, without incurring any uncertainties. On the other hand, a bi-directional tone alternation system involves uncertainty, increasing the complexity of the system, thus is harder to learn.

When we define the systems’ complexity on the basis of the number of features and on the level of the systems’ uncertainty, uni-directional systems are structurally simpler than bi-directional systems. If so, it could be hypothesized that uni-directional tone alternations (either left- or right-dominant) in which patterns agree in directionality will be learned more readily than bi-directional patterns where tones are preserved either on a left or on a right syllable.

### Directionality of Tone Alternations in Chinese

Work on Chinese phonology has reported various types of tone alternations (Yip, 1980; Yue-Hashimoto, 1987; Duanmu, 1993; Chen, 2000). Among those, tone sandhi is the most dominant pattern (Chen, 2000). Tone sandhi patterns are conditioned by adjacent tones or by prosodic or morphosyntactic environments and they involve either local substitution or extension (Zhang, 2007). For example, Mandarin has a local substitution tone sandhi system: when a syllable with the dipping-rising tone 214 precedes another 214 syllable, the first syllable’s original tone is replaced by the high-rising tone 35. Changzhou shows tone extension in its tone sandhi system. The tone on the first syllable is extended to the following tone. For instance, if the first syllable has a dipping tone 523, then a disyllabic word has a dipping melody 55–23, as a result of the first syllable’s tone extension (Zhang, 2007).

Huang and Do (2021) conducted a survey of tone sandhi patterns’ directionality and their phonological environments across 17 Chinese varieties from six dialectal groups (Northern, Wu, Min, Hakka, Xiang, and Jin), summarized in Table 1. Their survey included 17 varieties which cover a wide variety of geographic regions in China: the north (e.g., Beijing, Tianjin, Shanxi), northeast (e.g., Shandong), southeast (e.g., Shanghai, Fujian, Jiangsu), southwest (e.g., Sichuan, Yunnan) and central (e.g., Hunan, Henan) areas of China. A map of the dialects is provided (Supplementary Appendix A. A map of the 17 Chinese dialects). As Table 1 shows, among tone sandhi conditioned by adjacent tones, uni-directional tone sandhi patterns (e.g., Tianjin, Fuzhou, Changzhou) are more common than bi-directional ones (e.g., Huojia); and within uni-directional patterns, right-dominant patterns are more common than left-dominant ones. In Changzhou, Shanghai, and Tangxi, the left-dominant sandhi patterns are tone extensions, so the direction of left-dominant tone extension becomes rightward. Among tone sandhi conditioned by phonological environment and grammatical structure, tone sandhi patterns tend to be uni-directional within each grammatical category, with only one exception of Changsha. In other words, tone sandhi systems in Chinese varieties tend to show a certain directionality and usually it is rightward (right-dominant tone substitution and left-dominant tone extension) within a whole tone sandhi system or within each grammatical category, if a variety shows multiple directions.

**TABLE 1:** Tone sandhi directionality of 17 Chinese varieties.

Chinese dialects	Province/Area	Tone sandhi directionality	Tone sandhi environment
Beijing Mandarin	Beijing	Right-dominant	Tone sandhi is conditioned by phonological environment.
Tianjin	Tianjin	Right-dominant	
Boshan	Shandong	Right-dominant	
Kunming	Yunnan	Right-dominant	
Wuyi	Zhejiang	Right-dominant	
Xiamen	Fujian	Right-dominant	
Fuzhou	Fujian	Right-dominant	
Yudu	Jiangxi	Right-dominant	
Chengdu	Sichuan	Left-dominant	
Dongkou	Hunan	Left-dominant	
Changzhou	Jiangsu	Left-dominant	
Huojia	Henan	Bi-directional	
Shanghai	Shanghai	Right-dominant in one grammatical category, and left-dominant in another category	Tone sandhi is conditioned by phonological environment and grammatical structure.
Tangxi	Zhejiang	Right-dominant in one grammatical category, and left-dominant in another category	
Chaoyang	Guangdong	Right-dominant in one grammatical category, and left-dominant in another category	
Pingyao	Shanxi	Right-dominant in one grammatical category, and largely left-dominant in another category	
Changsha	Hunan	Bi-directional in each grammatical category	

### Tone Alternations in Mandarin^4^ and in Cantonese

In our experiments, participants’ native language is either Mandarin (Experiment 1) or Hong Kong Cantonese (Experiment 2). Both are tone languages, but their tone alternation patterns differ in terms of conditioning environments and directionalities. First, tone alternations in Mandarin are conditioned by adjacent tones, while tone alternations in Hong Kong Cantonese are triggered by morphological and semantic purposes (Matthews and Yip, 2011). Second, Mandarin tone alternations are generally right-dominant (Zhang, 2007), while tone alternations in Hong Kong Cantonese do not have a directionality asymmetry (Matthews and Yip, 2011). Details are provided below.

Mandarin Chinese has four citation tones: 55, 35, 214, 51, where 55 is a high-level tone, 35 is a high-rising tone, 214 is a dipping-rising tone, and 51 is a high-falling tone. There are three patterns of productive lexical tone alternations, which involve the third-tone sandhi (Zhang and Lai, 2010) and the fourth-tone sandhi (Lin, 1992), as shown in (1).

(1) Mandarin Chinese^5^

a. 214 → 35/__214
  xaw214 “good” – tɕju214 “wine” → xaw35-tɕju214 “good wine”
b. 214 → 21/__{55, 35, 51}
  xaw214 “good” – k^h^an51 “look” → xaw21-k^h^an51 “good-looking”
c. 51 → 53/__51
  fa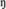 51 “release” – tɕja51 “holiday” → fa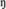 53-tɕja51 “have holidays”

As (1) shows, Mandarin tone sandhi is right-dominant: tone on the right syllable remains unchanged while tone on the left syllable undergoes alternation.^6^ Right-dominant tone substitution has phonetic grounding: Contour tones require longer duration, but the left syllable has relatively insufficient duration to carry contour tones. The final syllable of a prosodic unit is subject to lengthening and word-final lengthening has been well-documented (Oller, 1973; Beckman and Edwards, 1990, cited in Zhang, 2007). Therefore, reduction of a contour tone on the left syllable to a level tone or a contour with less radical pitch change are phonetically motivated.

Hong Kong Cantonese has six citation tones: a high level tone 55, a mid-level tone 33, a low level tone 22, a high rising tone 25, a low rising tone 23, and a low falling tone 21. Unlike Mandarin, Hong Kong Cantonese does not have tone sandhi. Tone alternation in Cantonese is generally referred to as tone change, because the alternation occurs due to morphological and semantic factors rather than phonological environment (Matthews and Yip, 2011). Tone change in Cantonese is associated with colloquial speech and generally does not occur in formal registers (Bauer and Benedict, 1997). The changes result in a high level tone 55 or a high rising tone 25 (Yue-Hashimoto, 1972). The changed tones are generally used to denote the nominalization of verbal actions or a familiar object (Yu, 2007). Different from Mandarin, tone change in Cantonese is largely unpredictable, because not all such words appear in changed tone and they must be listed in the lexicon (Yip, 2002). The main tonal change in Cantonese is in compounds and reduplications, and the changed tone can appear on the last syllable of a disyllabic word (Yue-Hashimoto, 1972), as shown in (2a–b). The changed tone may also appear in non-final positions, as in (2c–d).

(2) Hong Kong Cantonese^7^

The changed tone appears on the right syllable

a. jo25 “left” – yauh22 “right” → jo25-yau25 “about, approximately”
  Floating tone analysis of (a):

$$\begin{array}{ccccc} \text{jo}\; & \text{yauh}\; & \to \; & \text{jo}\; & \text{yau}\\ /\backslash \; & /|\backslash \; & & /\backslash \; & /\backslash \\ 2 5\; & 2 2<5>\; & & 2 5\; & 2 5 \end{array}$$

b. taai33 – taai33 → taai33 – taai25 “wife”
  The changed tone appears on the left syllable
c. si33 “try” – yat55 “one” – si33 “try” → si25-si33 “have a try”
  Floating tone analysis of (c):

$$\begin{array}{cccccc} \text{si}\; & <\text{yat}>\; & \text{si}\; & \to \; & \text{si}\; & \text{si}\\ /\backslash \; & /\backslash \; & /\backslash \; & & /\backslash \; & /\backslash \\ 3 3\; & 5 5\; & 3 3\; & & 2 5\; & 3 3 \end{array}$$

d. tihm21 “sweet” – yat55 “one” – tihm21 “sweet” → tim25-tihm21 “very sweet”

Cantonese tone change has been traditionally analyzed using a theoretical device of floating tone. Tone change patterns in (2a-b) could be understood as a floating high tone attaching to the end of the right syllable (Yip, 2002; Yu, 2007). As illustrated in the floating tone analysis of (a), the floating <5> docks onto the right syllable, resulting in a complex tone 225. Then the complex tone undergoes a simplification process by eliminating the medial tone segment, creating tone 25. Similarly, in tone change patterns (2c-d), as shown in the floating tone analysis of (c), the tone on *yat* is dissociated from the segmental melody and docks on the left syllable, forming a rising tone (Chen, 2000). In other words, the floating tone docks from the right to the preceding syllable in tone change patterns (2a-b) and (2c-d), which was regarded as right-dominant changes (Yip, 2002). Note that the “right-dominant” change in this context is different from the characterization of tone alternation directionality mentioned in section “Introduction”: the “right-dominant change” in the floating tone analysis means that the floating tone docks from the right to the preceding syllable in the *input* while the “right-dominant” in the tone directionality analysis indicates the position in the *output* that remains unchanged after tone alternation. Because the changed tone occurs either on the right syllable (patterns 2a-b) or on the left syllable (patterns 2c-d) in the output, Cantonese can be treated as having both left- and right-dominant tone alternations in terms of tone directionality. Crucially, different from Mandarin, Cantonese tone change is lexically restricted and does not have a systematic directionality asymmetry.

The target tone alternation pattern in our experiments is tone deletion. There were three reasons to choose tone deletion among various types of tone alternations. First, as previously mentioned, tone deletion is not attested in Mandarin or in Cantonese, which can minimize native language influence. Second, previous studies reported that tone sandhi patterns were not easily learnable in experimental settings (Zhang and Lai, 2010; Zhang, 2016; Zhang and Liu, 2016), which makes tone sandhi not an ideal testing site. For example, the nonce-probe tests conducted by Zhang (2016) showed that opaque Chinese tone sandhi patterns lacked full productivity and often failed to be applied to novel words. And even a number of fully productive sandhi patterns in the lexicon in Tianjin were not easily learnable by Tianjin speakers (Zhang and Liu, 2016). Third, evidence was provided that tone deletion patterns were learnable (Kao, 2017), which is different from the previous results on tone sandhi. Based on these considerations, we selected tone deletion as our test case. If the directionality found in tone sandhi or tone change patterns in L1 affects the learning of another type of tone alternation patterns, namely tone deletion, we expect that Mandarin speakers will learn uni-directional patterns better than bi-directional ones. On the contrary, Cantonese speakers will show no difference in learning left- and right-dominant patterns.

## Experiments

**FIGURE 1:**
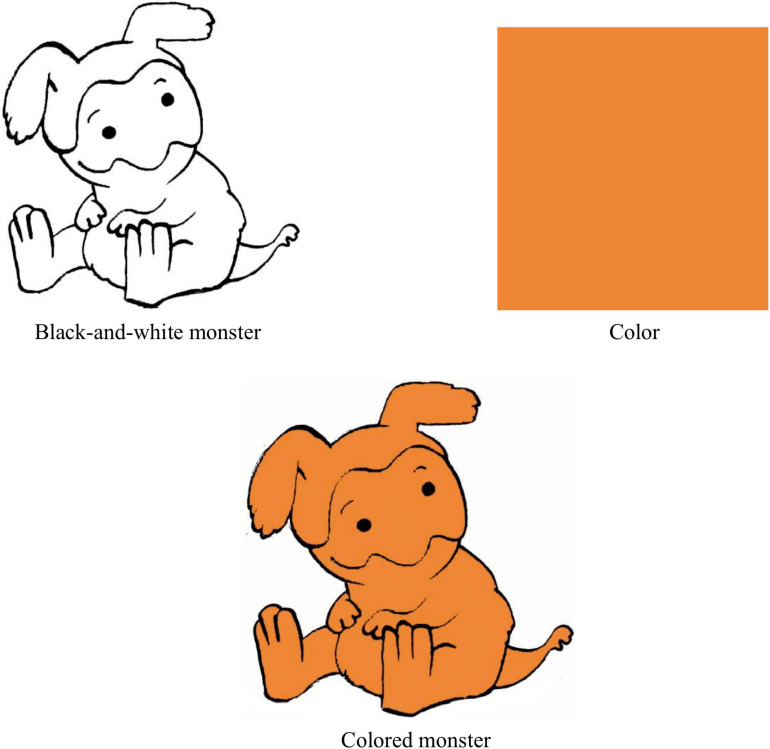
An example of visual stimuli.

We designed three artificial languages to investigate the role of structural bias in learning tone deletion (see Table 2). For each language, there were two target patterns and two filler patterns. Each target pattern showed a vowel fusion and a tone deletion, while each filler pattern showed a vowel fusion only without tone alternation. Language BD (bi-directional) is structurally complex in that the preserved tone after tone deletion is either on the left or on the right syllable. Languages LD (left-dominant) and RD (right-dominant) are simple in that the preserved tone is always on a single position. The visual stimuli were a black-and-white line drawing of a cartoon monster combined with a color creating a colored monster (see Figure 1 below).^8^ For the two target tone patterns across the three languages, pattern (a) was a rising tone 35 on the left syllable and a level tone 55 on the right syllable; pattern (b) was a falling tone 51 on the left syllable and a rising tone 35 on the right syllable. In Language BD pattern (a), for instance, the uncolored monster’s name was with a rising tone 35, and the color was with a level tone 55. The word for the colored monster then was with tone 55. In Language BD pattern (b), for instance, the uncolored monster’s name was with a falling tone 51, and the color was with a rising tone 35. The word for the colored monster then was with tone 51. In this way, an equal number of right-dominant and left-dominant tone patterns were created in Language BD, because this language exhibits bi-directional tone deletion patterns. For the two fillers, (c) and (d) of Language BD, tones on the left and right syllables were identical (either rising 35 or dipping-rising 214), thus no tonal alternation was exhibited among fillers. In Language LD, the two target tone patterns both preserved the tone on the left syllable, and were therefore uni-directional, and the fillers again had two syllables with the same tone (either level 55 or dipping-rising 214), and no tone alternation. In Language RD, the two target patterns both preserved the tone on the right syllable, and thus the alternation was again uni-directional; the fillers’ syllables were both falling 51 or dipping-rising 214, with no tone alternation. This design ensured that the resulting tones equally present level, rising, dipping-rising and falling tones across all three languages. Table 2 summarizes the stimuli design.

**TABLE 2:** Stimuli.

Language	Target tone patterns (26 items)		Fillers: no tonal alternation (33 items)	
BD (Bi-directional)	a. 35 + 55 → 55	b. 51 + 35 → 51	c. 35 + 35 → 35	d. 214 + 214 → 214
Examples	fu35 + an55 → fin55	ku51 + i35 → ki51	ta35 + i35 → ta35	pa214 + i 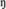 →214 → pa 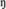 →214
LD (Left-dominant)	a. 35 + 55 → 35	b. 51 + 35 → 51	c. 55 + 55 → 55	d. 214 + 214 → 214
Examples	fu35 + an55 → fin35	ku51 + i35 → ki51	la55 + in55 → lan55	tu214 + an214 → tin214
RD (Right-dominant)	a. 35 + 55 → 55	b. 51 + 35 → 35	c. 51 + 51 → 51	d. 214 + 214 → 214
Examples	fu35 + an55 → fin55	ku51 + i35 → ki35	fu51 + i51 → fi51	pa214 + i214 → pa214

*55, 35, 214, 51 represent Mandarin level, rising, dipping-rising, and falling tones, respectively.*

*Audio stimuli: CV + V/VN (N: nasal) → CV/CVN.*

*Visual stimuli: black-and-white monster + color → colored monster.*

### Experiment 1

#### Stimuli

Experiment 1 was for Mandarin native speakers. For each language, 26 critical tone deletion patterns and 33 fillers (no tonal alternation) were created (Supplementary Appendix B. Stimuli). Table 3 illustrates the phonemes in each type of stimuli and their attestedness in Mandarin Chinese. As for the stimuli on the left syllable (CV structure), consonants were chosen from /w, f, k, m, n, p, t, l, s/ and vowels were from /u, a, i/. All the chosen phonemes were attested in Mandarin, to ensure that participants correctly perceive the phonemes in the stimuli. In order to match transitional probability, each consonant was equally combined with all three vowels. To match the consonants’ positional probabilities, the frequencies of all consonants in the target CV form were balanced. In the stimuli, the right syllable structure was either V or VN: /i, i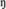, an, in/. The stimuli (CV and V/VN) before being combined conformed to Mandarin phonotactics. When an open syllable in CV form was followed by a syllable-initial vowel, the adjacent vowels across the syllable boundaries were combined into one, which takes the height of the left vowel and the backness of the right vowel. This is a typological resolution to vowel hiatus (Casali, 1996). For example, if a high back vowel /u/ was followed by a high front vowel /i/, the resulting vowel was /i/. Sometimes a resulting vowel was identical to an input vowel and the frequencies of preserving the left or the right vowel were balanced to avoid influence from the segments. For each language in the training phase, the segmental alternations were balanced: 18 items preserved the left vowel, 18 items preserved the right vowel, and 14 items created new vowels. In the testing phase, the segmental alternations were balanced as well: 10 items preserved the left vowel, 10 items preserved the right vowel, and 10 items had new vowels. For the 50 stimuli in CV/CVN structure (words for colored monsters) in the training phase, the frequencies of surface tones after alternation were balanced. For example, in Language BD, the 20 critical items had 10 level tones and 10 falling tones after tone alternation; the 30 fillers had 15 rising tones and 15 dipping tones after tone alternation. In the testing phase, the frequencies of the correct surface tones after alternation were balanced as well. For instance, in Language BD, the 16 critical items had eight level tones and eight falling tones after alternation, and the 14 fillers had seven rising tones and seven dipping tones after alternation.

**TABLE 3:** Stimuli attestedness in Mandarin.

	C	V	+	V/VN	→	CV/CVN
Stimuli	/w, f, k, m, n, p, t, l, s/	/u, a, i/		/i, i 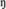 , an, in/		C+/i,a/+(/n, 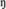 /)
Targets	Attested			Attested		Segment combinations are unattested
Fillers	Attested			Attested		Segment combinations are unattested
						Segment + Tone combinations are unattested
						Segments + Tone combinations are attested

The segmental combinations in the created target forms (CV/CVN structures) were unattested in Mandarin, to ensure that the phonological patterns in the artificial languages were different from those in the participants’ native language. The unattested segmental combinations are legal yet accidental gaps in Mandarin. The attestedness of fillers was in three types and the frequency of each type was identical: (a) the created segments were unattested in Mandarin; (b) the created segments were attested but their combinations with the tones were unattested; (c) the created segment and tone combinations were attested. The summary of the stimuli’s attestedness is given in Table 3. All stimuli were recorded by a native female speaker of Mandarin using an earset microphone and an Onyx Blackjack USB Recording Interface in a sound-proof booth at the authors’ institute. All monosyllables were recorded separately. For each item, the names of the uncolored monster, the color, and the colored monster were presented continuously to the participants. All stimuli underwent amplitude normalization using Praat (Boersma and Weenink, 2017).

#### Participants

Forty-nine adult native speakers of Mandarin (10 males, 39 females; age 18 or older) at the authors’ institute participated in the experiment and completed the task. No participants reported any speech or hearing disorders. Based on their self-report (Supplementary Appendix C. The language background questionnaire for Mandarin speakers), the participants’ dominant language was Mandarin. Of the 49 participants, 17 of them were monolingual speakers of Standard Mandarin. 32 of them had very limited knowledge of other Chinese varieties but none of them reported to have knowledge of Cantonese. Of the 49 participants, 16 of them learned Language BD; 16 learned Language LD; and 17 learned Language RD.

#### Procedure

The experiment took place in a sound-proof booth at the authors’ institute. A computer-based task was created through PsychoPy version 3.0 (Peirce et al., 2019). Before the experiment, the participants filled in a language background questionnaire to ensure that Mandarin was their dominant language. Because color is crucial in the materials, the participants also took a color blindness test (EnChroma Color Blind Test, 2020) and all of them passed the test. For the experiment, the participants sat in front of a computer and put on headphones and a lavalier microphone. The experiment consisted of three parts: an AXB test, a training phase, and a testing phase. For each participant, the stimuli were randomized within each phase. Neither orthography nor feedback were provided, making the experimental procedure an implicit learning task. The procedure was described to the participants in Mandarin.

The AXB test was used to assess the participants’ ability to distinguish between Mandarin level, rising, dipping-rising, and falling tones, all four of which were included in the experiment. They took six AXB tests presenting three sounds with the same segments, but with either the first or the third tone identical to the second tone (e.g., /mu35/, /mu35/, /mu51/) (Supplementary Appendix B. Stimuli). After the AXB test, the participants entered the training phase. They saw an instruction page that explained that they were going to learn an “alien” language and would be asked how to name colors and shapes in this language. Then, they went through two practice items (Supplementary Appendix B. Stimuli). For each item, they saw and heard the stimuli of an uncolored monster, a color, and a colored monster. To ensure the participants focused on the learning, they were asked to repeat after each audio file was played. After the practice session, the training phase consisting of 20 critical items and 30 fillers began. During the training, participants repeated the heard form of each colored monster’s name, and they were audio-recorded using a PMD661 MK2 digital recorder.

After the training, participants entered the testing phase, which consisted of 10 seen critical items from the training, six unseen critical items (the CV forms on the left syllable were unseen in the training), 11 seen fillers, and three unseen fillers (*n* = 30). The number of unseen critical items was relatively small,^9^ due to the extremely restricted Mandarin phonotactics. We created the maximum number of critical stimuli in which the left and right items (segment + tone combinations) were attested in Mandarin and Cantonese and the syllables after combination were unattested in Mandarin. Before the test, they were given two practice items, both without tone alternation (Supplementary Appendix B. Stimuli). Participants heard the names of an uncolored monster (e.g., /su35/) and a color (e.g., /i55/), and they were asked to choose the name of the colored monster. They chose from two options, both with the correct segments but with the two tonal options (one preserving the left tone and the other the right tone, such as /si35/ or /si55/). For fillers, the two tonal options presented the two tones appeared in fillers during the training. For example, for participants who learned Language BD, the two tonal options for fillers were tone 35 and tone 214. Half of the correct answers were given as the first option, and the other half as the second option. The two options were presented only auditorily.

#### Results

**FIGURE 2:**
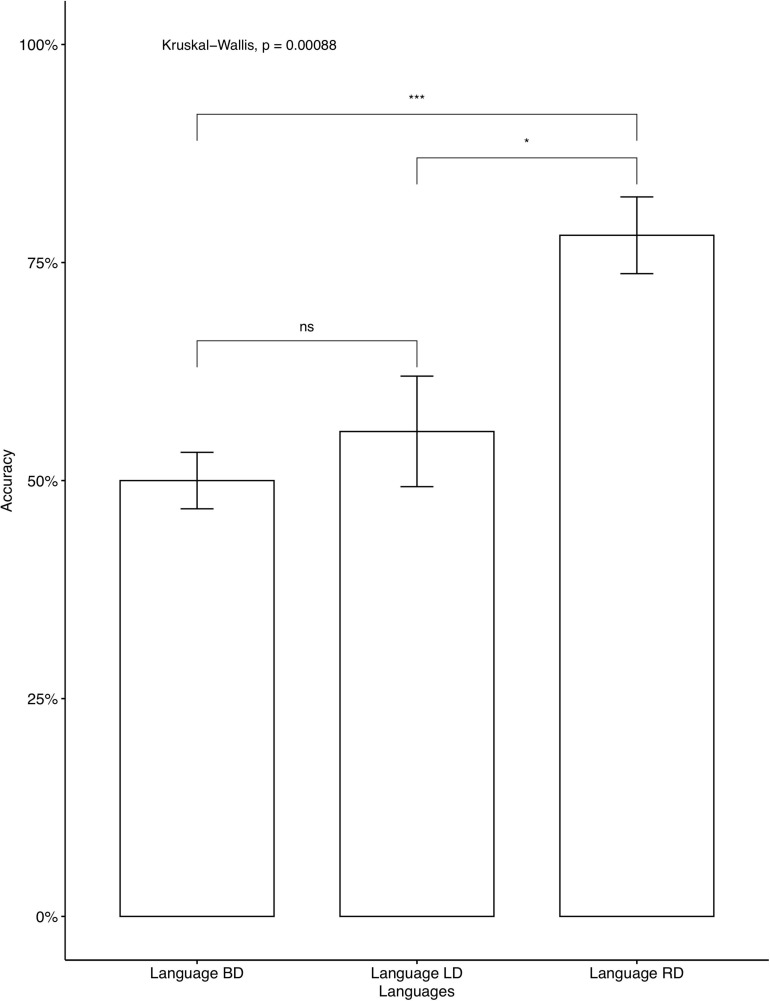
Accuracy on critical seen items: Mandarin native speakers. The 95% confidence interval is specified for each bar. *indicates significant mean difference, *p* < 0.05. ***indicates significant mean difference, *p* < 0.001.

**FIGURE 3:**
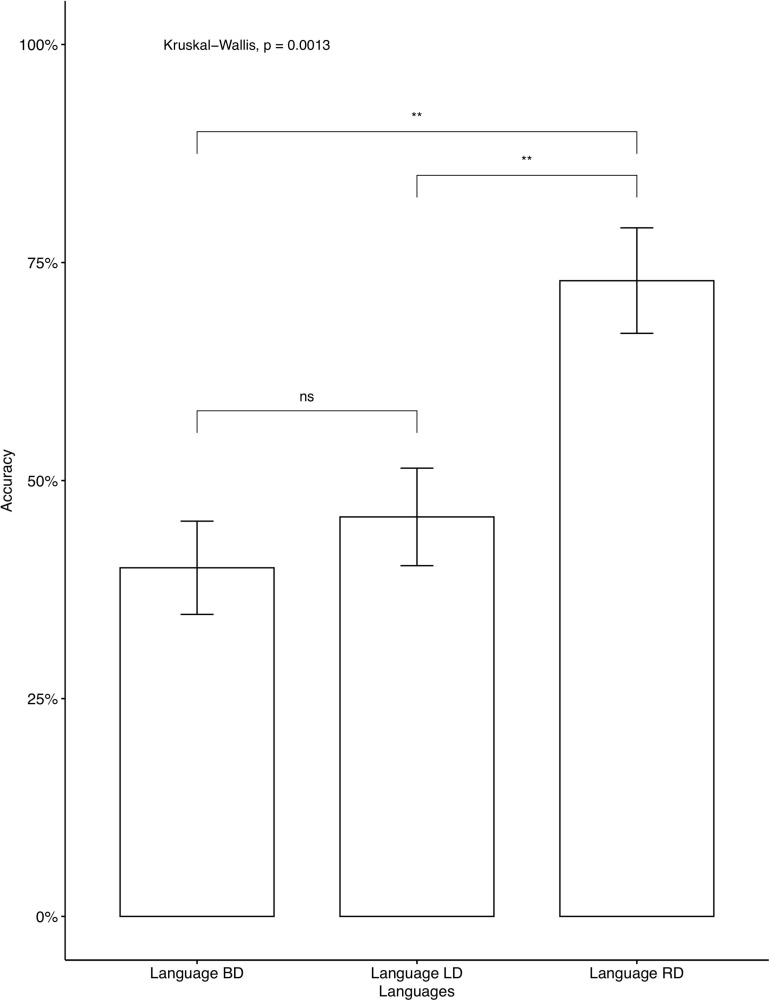
Accuracy on critical unseen items: Mandarin native speakers. The 95% confidence interval is specified for each bar. **indicates significant mean difference, *p* < 0.01.

**FIGURE 4:**
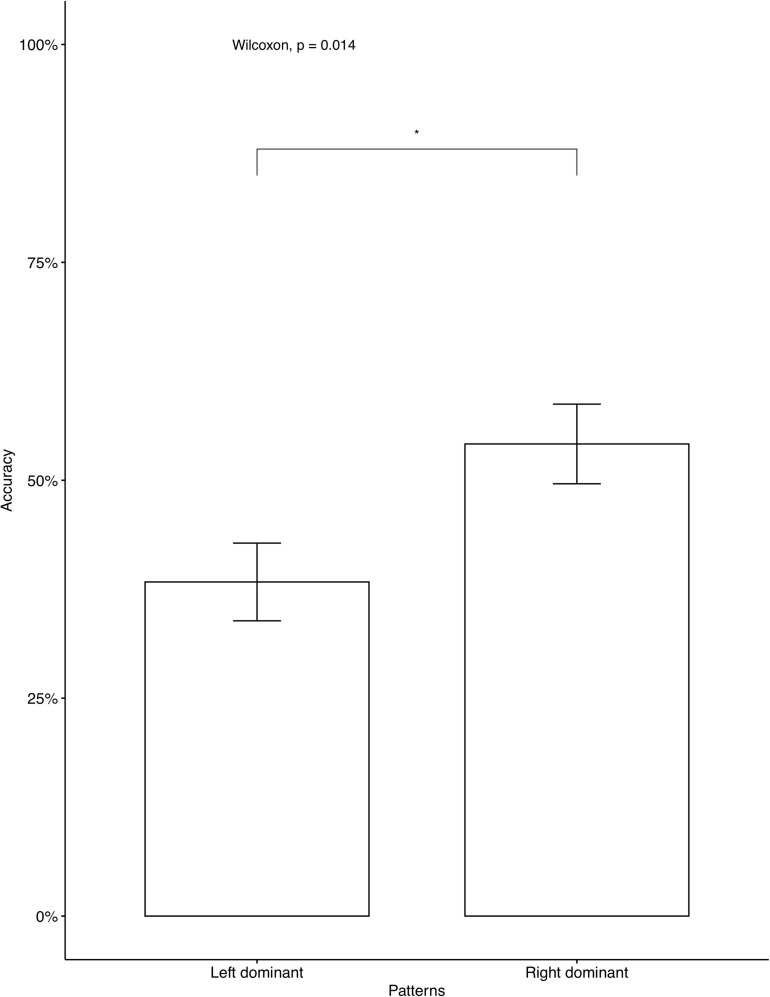
Accuracy on left dominant vs. right dominant patterns in Language BD. The 95% confidence interval is specified for each bar. *indicates significant mean difference, *p* < 0.05.

The average accuracy rates on the AXB task were very high (*M* = 0.979, *SD* = 0.056), which indicates that the participants were able to distinguish between the four contrastive tones. A phonetically trained Mandarin native speaker checked the recordings and confirmed that all participants correctly repeated the colored monsters’ names in the study phase, indicating that they focused on the learning. We analyzed individual accuracy rates on critical items and fillers separately. Two participants’ data were omitted, as their accuracy on fillers was below chance, which we interpreted as a lack of focus during the learning. The average accuracies on critical items for participants who learned Languages BD, LD, and RD were 46.25, 52, and 76.2%, respectively. A descriptive level seems to suggest that language RD was learned significantly better than Language BD and Language LD. The same tendency was seen in independent analyses conducted for the seen items (Figure 2) and for the unseen items (Figure 3). As Language BD has both left-dominant and right-dominant patterns, we checked learners’ performance independently for the right-dominant patterns and for the left-dominant patterns. As Figure 4 shows, the accuracy was significantly higher for the right-dominant patterns (54.17%) than left-dominant patterns (38.33%), which further suggests a bias toward right-dominant patterns. The average accuracies on fillers for participants who learned Languages BD, LD, and RD were 70, 80.80, and 83.93%, respectively.^10^ Language LD fillers were learned better than Language BD fillers [*t*(413.02) = 2.62, *p* < 0.01], and Language RD fillers were also learned better than Language BD fillers [*t*(400.38) = 3.47, *p* < 0.001]. No significant difference was found between Language LD and RD on fillers [*t*(443.84) = –0.87, *p* = 0.387]. These results indicate that participants who learned the two uni-directional languages (Language LD and Language RD) performed better on fillers than those who learned the bi-directional language (Language BD).

To test the statistical significance of the results, a logistic regression was performed using the glmer function of the lme4 package (Bates et al., 2015) in R (R Core Team, 2018). The responses on critical items of the binary-forced-choice task were converted to binary values (0 = incorrect response; 1 = correct response). Two sum coded factors, Simplicity (Uni-directional vs. Bi-directional) and Directionality (Neutral vs. Left-dominant vs. Right-dominant), were our interests. Note though that the two factors are not independent, in that bi-directional patterns were always neutral in directionality. To address this issue, a new categorical variable which merges Simplicity and Directionality was assumed as an independent factor. The new categorical variable has three levels, namely “Bi-directional: neutral,” “Uni-directional: left-dominant,” and “Uni-directional: right-dominant.” In this way, the model allowed us to directly read the two contrasts we are interested in, namely Bi-directional vs. Uni-directional and Left-dominant vs. Right-dominant. Random intercepts were included for Items and Participants, and a random slope for the merged Simplicity and Directionality by Participants. The results in Table 4 show that the accuracy rate for the Bi-directional language was significantly lower than the Uni-directional languages (Language LD and Language RD) (β = –0.8852, *p* < 0.001). It further shows that participants’ performance was significantly worse in the Left-dominant language, compared to the Right-dominant language (β = –1.2535, *p* < 0.001).

**TABLE 4:** The results of a logistic regression model for the response accuracy among Mandarin speakers.

	Estimate	*SE*	*z*-value	*p* (*z*)
Intercept	0.4351	0.1506	2.890	<0.01**
Bi-directional vs. Uni-directional	–0.8852	0.2394	–3.698	<0.001***
Left-dominant vs. Right-dominant	–1.2535	0.3793	–3.305	<0.001***

**indicates significant mean difference, p < 0.01. ***indicates significant mean difference, p < 0.001.

An additional analysis of pairwise comparisons was conducted for the three levels using the emmeans package (Lenth et al., 2020). The results are in Table 5. As shown, the accuracy rate difference between Language BD (Bi-directional: neutral) vs. Language LD (Uni-directional: left-dominant) was not significant (*p* = 0.6629), while the differences were significant between Language BD (Bi-directional: neutral) vs. Language RD (Uni-directional: right-dominant) (*p* < 0.001) and between Language LD (Uni-directional: left-dominant) vs. Language RD (Uni-directional: right-dominant) (*p* < 0.01), confirming our observation in Table 4.

**TABLE 5:** Pairwise comparisons of merged simplicity and directionality.

Contrast	Odds ratio	*SE*	*z* ratio	*p*-value
Bi-directional: neutral vs. Uni-directional: left-dominant	0.772	0.2309	–0.864	0.6629
Bi-directional: neutral vs. Uni-directional: right-dominant	0.220	0.0687	–4.851	<0.001***
Uni-directional: left-dominant vs. Uni-directional: right-dominant	0.286	0.1083	–3.305	<0.01**

**indicates significant mean difference, p < 0.01. ***indicates significant mean difference, p < 0.001.

#### Discussion

The results of Experiment 1 showed a learning bias toward uni-directional patterns, supporting our hypothesis. In other words, the learning of tone alternations was biased toward structurally simpler patterns. However, crucially, only the right-dominant patterns, not left-dominant ones, showed better learning performance than bi-directional ones. Additionally, as Figure 4 shows, right-dominant patterns were learned significantly better than left-dominant patterns in Language BD, which further supports the bias toward right-dominant alternations.

A simple structural bias cannot account for the current results, because the learning outcomes between the left-dominant and right-dominant patterns were not equivalent. A question is then what factors facilitated the learning of the right-dominant pattern. Right dominance in tone deletion has its phonetic grounds. Recall that our critical stimuli had either rising or falling tone on the left syllable. The left syllable has relatively insufficient duration, thus a contour tone on the left syllable tends to undergo changes (Zhang, 2007): In producing contour tones, the acoustic change is made by a single articulator, the vocal folds. Vocal fold tension changes result from laryngeal muscle contraction and relaxation, which must be sequenced to produce the pitch variation in contour tones. While a complex segment can be produced with overlapping oral constrictions, a contour tone needs enough duration to be produced (Zhang, 2002). As to the perception of contour tones, the sequenced stretching or relaxation of the vocal folds requires a greater duration of its carrier (Zhang, 2002). For a syllable’s contour tone bearing ability, the non-final syllables have relatively insufficient duration to carry contour tones in comparison to the final syllable, because the final syllable of a prosodic unit is subject to lengthening and word-final lengthening has been well-documented (Oller, 1973; Beckman and Edwards, 1990, cited in Zhang, 2007). Typological studies among African languages, Oto-Manguean languages and Sino-Tibetan languages have found that contour tones are asymmetrically distributed toward domain-final positions (Clark, 1983; Zhang, 2002). Thus, considering insufficient ability of the left syllable to carry contour tones in the input, the left tone in the input is less stable in comparison to the right tone, thus is more likely to be a target of deletion. If so, our results may suggest that the simplicity of tone alternation directionality affects learning (better learning of uni-directional patterns than bi-directional patterns), but the nature of this simplicity is phonetically grounded, as evidenced by the asymmetry of the learning of left-dominant and right-dominant patterns. A mere substantive bias does not explain the current results either. Language BD exhibited half of phonetically natural patterns while Language LD showed only unnatural patterns. If substantive bias alone had played a role in learning, the accuracy of Language BD should have been higher than that of Language LD, while the results showed no supporting evidence for that. Moreover, the average accuracy rate on the right-dominant patterns in Language RD (76.17%) was significantly higher than the average accuracy rate on the right-dominant patterns in Language BD (54.17%) [*t*(203.02) = 4.16, *p* < 0.001], which further suggests the effect of structural bias. Therefore, it is a phonetically grounded structural bias that played a role in learning tone alternation patterns in our study.

An alternative explanation can be pursued that the current results are purely due to perceptual salience differences. This analysis specifically relies on the fact that perceptual salience between the left and right vowels in the input differ due to their different durations. Recall that there were two types of segmental alternation patterns in the experiment: (1) CV_1_ + V_2_ → CV and (2) CV_1_ + V_2_N → CV. As to the vowel durations, the duration of the vowels in CV_1_ form is shorter than that of vowels in V_2_ or V_2_N forms due to their position where CV_1_ is followed by an adjacent vowel-initial syllable. Note that we did not synthesize the duration of syllables in order to make the languages sound natural. Therefore, it is conceivable that the longer vowel duration on the right syllable, V_2_ or V_2_N forms, might have provided higher perceptual salience on the right side, thus had triggered the right-dominant preference. If this was the case, we also expect that the vowel duration cue had contributed to distinguish participants’ performance in other phonological positions as well. When the durations of V_2_ in the two types of second syllables are considered, i.e., V_2_ and V_2_N, the duration of the vowels in the open syllable V_2_ should be overall longer than that of the vowels in the closed syllable V_2_N, because of a coda in V_2_N. So, if the duration of vowels had affected the results due to perceptual salience differences, the average accuracy rates on the processes (1) CV_1_ + V_2_ and (2) CV_1_ + V_2_N should be different. To check this possibility, we compared the accuracy rates for (1) and (2) within each language. For Language BD, the average accuracy rates on critical items for processes (1) and (2) were 57.33 and 54.74%, respectively [*t*(601.18) = 0.69, *p* = 0.491], showing no difference. For Language LD, the average accuracy rates on critical items for processes (1) and (2) were 65.42 and 64.69%, respectively [*t*(681.22) = 0.21, *p* = 0.833], again showing no significant difference. For Language RD, the results remain the same: the average accuracy rates on critical items for processes (1) and (2) were 79.79 and 78.13%, respectively [*t*(590.2) = 0.55, *p* = 0.585]. This result suggests that it is not possible to account for the current results purely relying on acoustic cues, such as vowel duration. Furthermore, one of the two tone deletion patterns in the experiment was a rising tone on CV_1_ while it was a level tone on V_2_/V_2_N. Given that a rising tone has intrinsically longer duration than a level tone (Zhang, 2002), at least the half of the deletion patterns had longer tonal durations on the left syllables. Therefore, it further argues against a possibility that the right-dominant preference was due to the longer vowel duration on the right syllables. In addition, for all critical items, the duration of the right syllables (*M* = 0.625 s) was not significantly longer than the duration of the left syllables (*M* = 0.597 s) [*t*(30.231) = 1.24, *p* = 0.2247]. When we categorized the critical items into processes (1) CV_1_ + V_2_ and (2) CV_1_ + V_2_N and compared the duration within each process, the same tendency was true: For the CV_1_ + V_2_, the duration of the right syllables (*M* = 0.651 s) was not significantly longer than the duration of the left syllables (*M* = 0.602 s) [*t*(8.0827) = 1.142, *p* = 0.2861]. Also for the CV_1_ + V_2_N, the duration of the right syllables (*M* = 0.613 s) was not significantly longer than the duration of the left syllables (*M* = 0.595 s) [*t*(19.725) = 0.69332, *p* = 0.4962]. Therefore, it suggests that the pure durational cue itself cannot account for the right-dominant preference.

Note though that a possible source of the right-dominant bias may still be not phonetically grounded structural bias, but rather a first language influence, because Mandarin tone sandhi patterns are generally right-dominant (Zhang, 2007). Although tone sandhi is different from tone deletion, it is still plausible that knowledge about directionality from one tone alternation pattern could affect the learning of another tone alternation pattern. Therefore, we conducted Experiment 2 to see whether the results in Experiment 1 were driven by a phonetically grounded structural bias or were a simple reflection of a native language transfer effect.

### Experiment 2

Experiment 2 was conducted with adult Cantonese native speakers. Because Mandarin is widely used in Hong Kong (Leung and Wong, 1996), it is practically not possible to find speakers in Hong Kong who have zero exposure to Mandarin. Thus, we chose our participants with bare or very low levels of Mandarin knowledge. The 31 participants (10 males, 21 females) consisted of native speakers of Hong Kong Cantonese aged over 18. In the recruitment advertising, we stated that Hong Kong Cantonese speakers with only bare knowledge of Mandarin and other Chinese varieties were eligible to participate. Before the experiment, participants filled in a self-rating scale (Supplementary Appendix D. Self-rating scale for Cantonese speakers’ Mandarin proficiency) about their Mandarin proficiency. All participants rated themselves Level 2 or below on six levels (Level 0–Level 5 in the self-rating scale), the levels of which show no communicative proficiency in Mandarin. No participant reported any speech or hearing disorder. To examine whether speakers without tone alternation conditioned by adjacent tones and directionality asymmetry in L1 still prefer right-dominant alternations, the participants learned either Language LD (left-dominant) or Language RD (right-dominant). Of the 31 participants, 15 of them learned Language LD and 16 learned Language RD. All the phonemes in the stimuli are also attested in Cantonese, thus the Cantonese native speakers were able to perceive the phonemes. For each language, the stimuli (CV and V/VN) before combination conform to Cantonese phonotactics.^11^ After combining the left and right syllables, 22 out of 59 segmental combinations in the created forms (CV/CVN structures) of each language are unattested in Cantonese.^12^ Specifically, for critical items, 14 out of 26 segmental combinations are unattested in Cantonese. The high-level tone and high-rising tone in our stimuli are attested in Cantonese, and dipping-rising and falling tones are unattested in Cantonese. The experiment’s procedure was described to the participants in Cantonese. Other procedures are the same as those of Experiment 1.

#### Results

**FIGURE 5:**
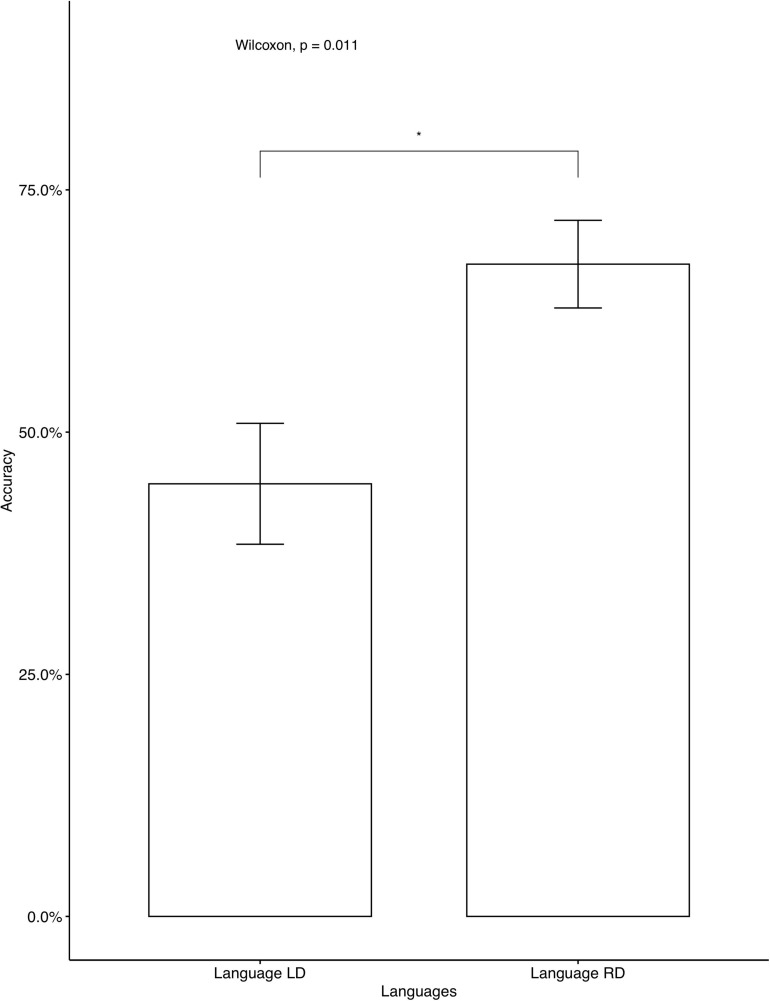
Accuracy on critical seen items: Cantonese native speakers. The 95% confidence interval is specified for each bar. *indicates significant mean difference, *p* < 0.05.

**FIGURE 6:**
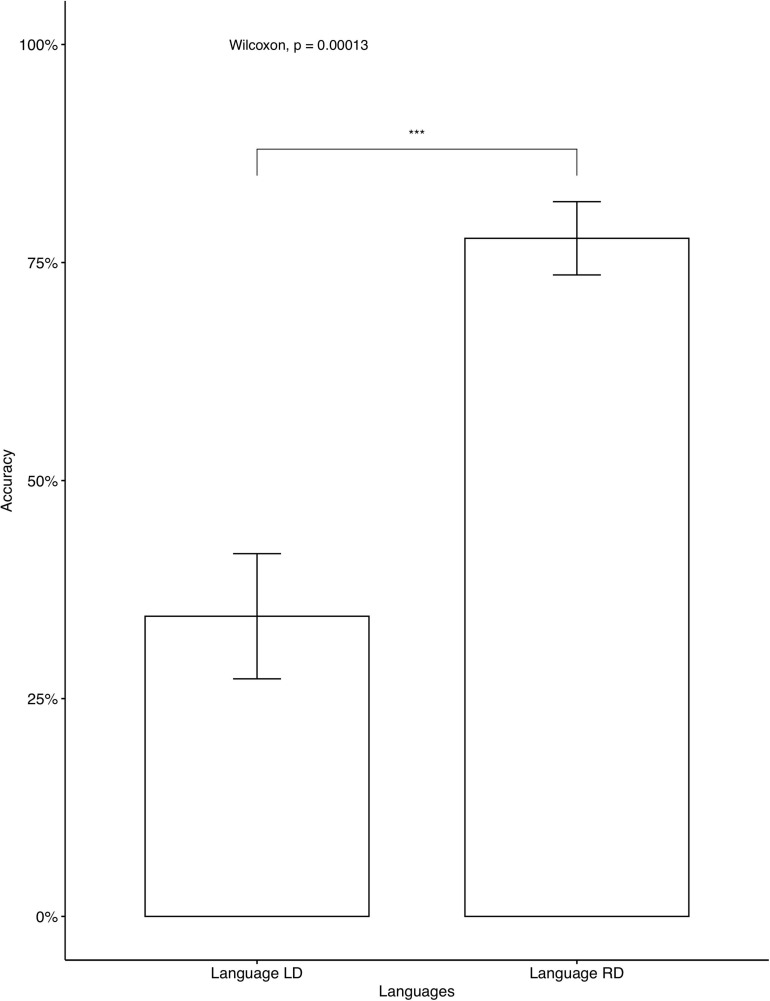
Accuracy on critical unseen items: Cantonese native speakers. The 95% confidence interval is specified for each bar. ***indicates significant mean difference, *p* < 0.001.

In the AXB test, the participants reached a very high accuracy (*M* = 0.939, *SD* = 0.102), which indicates that the Cantonese speakers correctly distinguished between the four different tones. The recordings from the training phase showed that all participants correctly produced the colored monsters’ names, indicating that they focused on the learning. We then examined individual accuracy rates on fillers and critical items separately. One participant’s data were omitted, as the accuracy for fillers was below chance. All other participants’ data were entered into the analysis. For the average accuracy rates on all critical items, Language RD (accuracy = 71.25%) was learned significantly better than Language LD (accuracy = 40.83%). The tendency was consistent when separate analyses were conducted for seen items (Figure 5) and for unseen items (Figure 6). The average accuracies on fillers for participants who learned Language LD and Language RD were 78.57 and 80%, respectively^13^ and no significant difference was found [*t*(417.73) = –0.36, *p* = 0.719].

A logistic regression was constructed with random intercepts for Participants and Items. It assumed the Directionality factor with two levels (Left-dominant vs. Right-dominant) to test whether right-dominant patterns were learned better than left-dominant ones. The results, presented in Table 6, revealed a significant effect of Directionality: right-dominant patterns were learned better than left-dominant ones (β = 1.4438, *p* < 0.001).

**TABLE 6:** The results of a logistic regression for the response accuracy among Cantonese speakers.

	Estimate	*SE*	*z* value	*p* (*z*)
Intercept	–0.4426	0.2466	–1.795	0.0727.
Right-dominant	1.4438	0.3535	4.084	<0.001***

***indicates significant mean difference, p < 0.001.

#### Discussion

The results of Experiment 2 suggest that the right-dominant bias observed in Experiment 1 was not purely due to the first language influence. If so, the results suggest that a structural bias plays a role in tone alternation learning, as evidenced by the better performance in learning uni-directional, right-dominant tone alternations than bi-directional alternations. Crucially, the structural bias should be phonetically grounded, as evidenced by the better learning outcomes for right-dominant alternations than left-dominant alternations by both Mandarin (Experiment 1) and Cantonese (Experiment 2) speakers. The results from both experiments are consistent with the survey data of 17 Chinese dialects presented in Table 1. As mentioned in section “Directionality of tone alternations in Chinese,” either throughout a tone sandhi system or within each grammatical category, uni-directional tone alternations were more common than bi-directional ones, and rightward patterns were more common than leftward ones within uni-directional patterns.

While the current results cannot be attributed solely to the first language influence, there is still room to navigate a potential influence of native languages. When the average accuracies in Language LD (left-dominant) and RD (right-dominant) by Mandarin and Cantonese speakers were compared, Mandarin speakers scored higher than Cantonese speakers in Language LD [*t*(492.84) = 2.49, *p* = 0.013] while no significant difference was found between Mandarin and Cantonese speakers in learning Language RD [*t*(486.39) = 1.24, *p* = 0.215], suggesting a native language effect. It is plausible to think that Mandarin speakers might have found the artificial language easier to learn than Cantonese speakers, because tone alternation patterns in Mandarin are similar to the target patterns of this experiment, but not in Cantonese: Mandarin has tone alternations conditioned by adjacent tones while Cantonese tone alternation is conditioned by morphological factors. Mandarin speakers’ better performance could also be attributed to the different familiarity level of the four tones for Mandarin and Cantonese speakers. While all the tones are familiar for Mandarin speakers, the dipping-rising and falling tones in our stimuli are unattested in Cantonese. The high-level tone and high-rising tone are attested in Cantonese, although the phonetic details of the exact pitch contours differ between the two languages. Therefore, the tones and tone alternation types are more familiar for Mandarin speakers. If native language had influenced participants’ performance in the artificial language learning setting, then why such effect was found only in the learning of Language LD but not in Language RD? Our results suggest that in learning phonetically grounded simple tone alternations (Language RD), L1 evidence did not lead to significant learning differences but in learning phonetically ungrounded tone alternation patterns (Language LD), L1 evidence did facilitate the learning. Such tendency echoes the findings from Baer-Henney et al. (2015) whereby the acquisition of “uncertain” patterns involving more variation and less training was boosted more by a substantive bias rather than L1, while the influence of L1 was stronger in the acquisition of patterns with “higher certainty” which involved less variation and longer training. Regarding the current results, L1 evidence facilitated Mandarin speakers’ learning especially when the pattern lacked phonetic substance (Language LD). However, such L1 effect was not clear when the pattern was grounded on phonetic substance (Language RD), which was presumably equally available to Mandarin and Cantonese speakers.

Similar to the results in Experiment 1, no accuracy difference was found between the segmental processes (1) CV_1_ + V_2_ and (2) CV_1_ + V_2_N. For Language LD, the average accuracy rates on critical items for processes (1) and (2) were 58.44 and 58.46%, respectively [*t*(368.18) = –0.004, *p* = 0.997], showing no significant difference. For Language RD, the average accuracy rates on critical items for processes (1) and (2) were 75.33 and 70.26%, respectively [*t*(349.43) = 1.31, *p* = 0.189], again showing no significant difference. Therefore, the result further suggests that the vowel duration was not a significant cue to contribute to the right-dominant preference. Recall that 14 out of 26 critical segmental combinations were unattested in Cantonese. The average accuracy rates were not significantly different depending on the attestedness of segmental combinations in both Language LD [*t*(214.87) = 0.05, *p* = 0.9582] and Language RD [*t*(217.56) = 0.98, *p* = 0.3276], suggesting no crucial role of the attestedness of segmental combinations in learning tone deletion patterns.

Regarding the effect of morphological constructions, the uncolored monster name was the head of a noun phrase in our stimuli, and the color name was the noun phrase’s modifier. In natural language, tone deletion tends to be applied to non-head positions (Yip, 2007). For example, in Shanghai, tone is deleted if it is not on the head syllable of a word. In our experiments, the heads (uncolored monster names) were on the left syllable, and so the left tone would be more likely to be preserved if morphological structure plays a role. However, our results showed the opposite trend, which further supports the effect of the phonetically grounded structural bias in learning tone alternations, as opposed to a morphological effect.

## Discussion and Conclusion

Our experiments confirmed the role of structural simplicity in the directionality of tone deletion, as evidenced by the better learning of uni-directional, right-dominant deletions than bi-directional ones by Mandarin native speakers. Crucially, within uni-directional deletions, right-dominant patterns were learned better than left-dominant patterns both by Mandarin and by Cantonese speakers. The tendency to preserve the tone on the right syllable over the left syllable cannot be attributed to a pure L1 transfer effect, because tone alternations conditioned by adjacent tones are unattested in Cantonese. The preference for right-dominant tone deletions can be directly explained by its phonetic grounds, because the left syllable does not provide sufficient grounds to carry contour tones, and therefore input tones on the left syllable are less stable and are more likely to be deleted. Therefore, the current results suggest that structurally simple tone deletions grounded on phonetic factors are learned best. Recall that our critical stimuli had contour tones on the left syllable, in order to explore the effect of phonetic underpinning on tone alternation learning. It remains to be seen if there is a right-dominant bias when level tones appear on the left syllable, the pattern of which lacks a similar phonetic motivation. Given the current finding, we expect that learning outcomes will not be biased toward right-dominant patterns when the phonetic motivation is lacking. Besides, future work could test a population with zero exposure to Mandarin, to investigate whether there is difference between different populations.

The relationship between structural simplicity and phonetic substance has been discussed in the literature on phonological learning biases. First, work in segmental phonology has suggested that the two biases are frequently intertwined. For example, White (2014) compared the learning of saltatory alternations, such as [p ∼ v] and non-saltatory alternations, such as [b ∼ v]. White reported that participants who were trained on saltatory alternations changed intermediate sounds at a high rate in the testing, although there was no evidence for such changes in the input. The tendency to avoid saltatory alternations can be attributed to both substantive and structural biases. In terms of phonetic motivation, saltation avoidance can be explained by the principle of perceptual minimal modification (Steriade, 2008). Regarding structural simplicity, saltatory alternations involve more featural changes than non-saltatory ones. Similarly, in the study by Skoruppa et al. (2009), the better learning of the alternation [p ∼ t] than [p ∼ s] or [p ∼ z] can be attributed to both the number of featural changes and perceptual similarity.

Despite the frequent intertwining of structural and substantive factors, it seems that structural simplicity is stronger than the substantive bias effect, as previous studies on learning biases in segmental phonology have provided robust evidence for structural bias and mixed results for substantive bias (see Moreton and Pater, 2012a,b, for summaries). We believe that our findings provide an additional angle to consider a relation between structural and phonetic components in phonological learning. Recall the learning outcomes of Language LD. This language is structurally simple (uni-directional tone alternations) but the directionality lacks a phonetic motivation. The overall learning performance of this language was significantly lower than that for Language RD (right-dominant). This suggests that structural simplicity becomes “relevant” to learning when the structural components are phonetically grounded. Although phonetic naturalness may not have a privileged role on its own in synchronic grammar (Ohala, 1974), and substantive bias is arguably weaker than structural bias, our results suggest that phonetic substance may facilitate the accessibility of structural simplicity in learning.

Finally, we note that the structural bias found in our experiments may not necessarily be phonology-specific. Instead, the preference for uni-directional patterns can be attributed to a general structural bias, beyond linguistic patterns. As mentioned in section “Defining Structural Complexity of Tone Alternations’ Directionality,” bi-directional tone alternations are more complex than uni-directional ones when considerations from non-linguistic pattern learning and the level of (un)certainty are taken into account. Therefore, it still remains to be seen to what extent the structural bias we have observed in tone deletion learning should be attributed to a phonological learning bias and how much of it is rooted in a general structural bias.

## Data Availability Statement

The raw data supporting the conclusions of this article will be made available by the authors, without undue reservation.

## Ethics Statement

The studies involving human participants were reviewed and approved by the Human Research Ethics Committee, The University of Hong Kong. The patients/participants provided their written informed consent to participate in this study.

## Author Contributions

TH and YD were contributed to the conceptualization, methodology, data analysis, and writing, reviewing, and editing. TH was responsible for the data collection. Both authors contributed to the article and approved the submitted version.

## Conflict of Interest

The authors declare that the research was conducted in the absence of any commercial or financial relationships that could be construed as a potential conflict of interest.

## Publisher’s Note

All claims expressed in this article are solely those of the authors and do not necessarily represent those of their affiliated organizations, or those of the publisher, the editors and the reviewers. Any product that may be evaluated in this article, or claim that may be made by its manufacturer, is not guaranteed or endorsed by the publisher.

## Footnotes

1 Tones in this paper are transcribed in the Chao tone letter system, where “1” represents the lowest and “5” represents the highest pitch (Chao, 1948).

2 In this paper, T represents any tone of the dialect.

3 The realization of Mandarin final neutral tone has been sometimes analyzed as a tone deletion (e.g., Yip, 1980) while others analyzed the pattern as a type of tone sandhi (Lin, 1992; Zhang, 2007). Note that the neutral tone was not included in the experiment, thus a direct transfer effect from the pattern, even if it’s assumed as tone deletion, is not relevant here.

4 “Mandarin” refers to the standard variety of Mandarin spoken in Mainland China.

5 Real world examples of Mandarin tone sandhi are from Zhang and Lai (2010); they are transcribed in IPA.

6 Note that there are exceptions: final neutral tones are realized with left-dominant sandhi. Certain word-final morphemes in Mandarin are reduced and carry a neutral tone. Neutral tone itself does not have a pitch value, deriving it from the previous tone. In disyllabics, the neutral tone is 41 after tone 55; it is 52 after tone 35; it is 33 after tone 214; and it is 21 after tone 51 (Lin, 1992). Another exception is the third tone in certain reduplicated adjectives with left-dominant sandhi. For example, in xaw-xaw-ɚ “good, well,” the pattern is 214-214 → 214-55 (Yip, 1980).

7 Real world examples of Cantonese tone change are from Matthews and Yip (2011); they are transcribed in the Yale system.

8 The monsters were originally created by van de Vijver and Baer-Henney (2014).

9 The number of unseen items in some artificial language learning experiments are also relatively small, for example, Gong et al. (2016) used eight unseen items.

10 Regarding average accuracies on seen and unseen fillers in Experiment 1, seen fillers were learned better than unseen fillers in Language LD [*t*(64.965) = 2.13, *p* < 0.05] and Language RD [*t*(59.782) = 2.87, *p* < 0.01]. No significant difference was found between seen and unseen fillers in Language BD [*t*(70.188) = –0.18, *p* = 0.855]. For average accuracies on the three types of filler attestedness, no significant difference in learning the three types of fillers was found in Languages LD and RD. For Language BD, participants learned the unattested fillers (the segments whose combinations were unattested) better than the attested fillers (the segment and tone combinations were attested) [*t*(98.732) = 3.02, *p* < 0.01].

11 There are two exceptions: (a) the CV form /tu/ is unattested in Cantonese; (b) the VN form /i/ is realized as [I] in Cantonese.

12 /wi/, /win/, /fin/, /fa/, and all the CVN forms with /i/ are unattested segmental combinations in Cantonese.

13 For seen and unseen fillers in Experiment 2, seen fillers were learned better than unseen fillers in Language LD [*t*(60.55) = 2.25, *p* < 0.05]. No significant difference was found between seen and unseen fillers in Language RD [*t*(66.131) = 0.89, *p* = 0.374].
