# Supplementary material for: Phonetically Grounded Structural Bias in Learning Tonal Alternations
Source: Front Psychol. 2021 Jul 26;12:705766. doi: 10.3389/fpsyg.2021.705766 (PMC8350328; doi:10.3389/fpsyg.2021.705766)
Supplement: Supplementary file 1 [file Data_Sheet_1.pdf]

Appendix A. A map of the 17 Chinese dialects

The dialects in our survey are circled in blue.

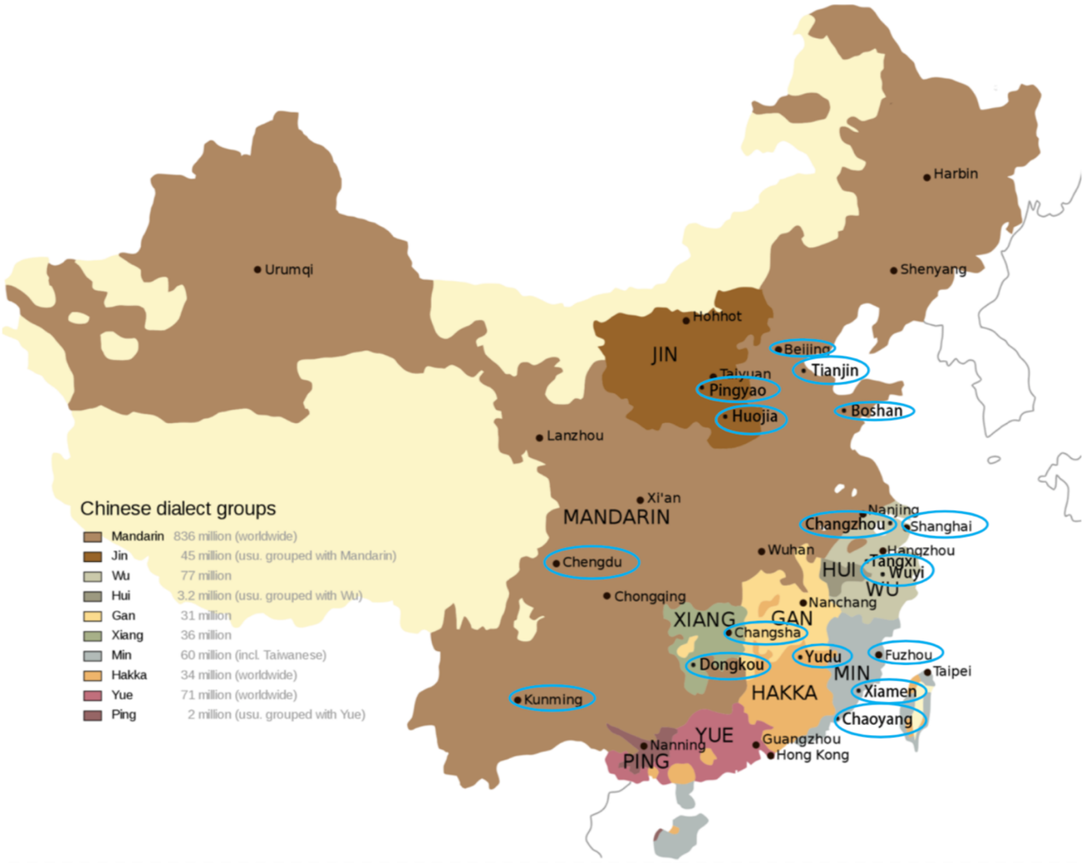

Appendix B. Stimuli[1]

AXB test

	A	X	B
1	su55	su55	su35
2	wa214	wa55	wa55
3	ta51	ta51	ta55
4	faŋ214	faŋ35	faŋ35
5	mu35	mu35	mu51
6	lan51	lan214	lan214

Practice items for the training phase

Language BD				Language LD				Language RD			
	CV	V/VN	CV/CVN		CV	V/VN	CV/CVN		CV	V/VN	CV/CVN
1	pu214	iŋ214	piŋ214	1	pu214	iŋ214	piŋ214	1	pu214	iŋ214	piŋ214
2	mu35	an35	min35	2	li55	an55	lin55	2	li51	an51	lin51

Training phase—critical items[2]

Language BD				Language LD				Language RD			
	CV	V/VN	CV/CVN		CV	V/VN	CV/CVN		CV	V/VN	CV/CVN
1	wu35	i55	wi55	***1***	wu35	i55	wi35	***1***	wu35	i55	wi55
2	wu51	i35	wi51	***2***	wu51	i35	wi51	***2***	wu51	i35	wi35
3	wu35	an55	win55	***3***	wu35	an55	win35	***3***	wu35	an55	win55
4	wu51	an35	win51	***4***	wu51	an35	win51	***4***	wu51	an35	win35
5	wu35	iŋ55	wiŋ55	5	wu35	iŋ55	wiŋ35	5	wu35	iŋ55	wiŋ55
6	wu51	iŋ35	wiŋ51	6	wu51	iŋ35	wiŋ51	6	wu51	iŋ35	wiŋ35
7	fu35	i55	fi55	7	fu35	i55	fi35	7	fu35	i55	fi55
8	fu51	i35	fi51	8	fu51	i35	fi51	8	fu51	i35	fi35
9	fu35	an55	fin55	9	fu35	an55	fin35	9	fu35	an55	fin55
10	fu51	an35	fin51	10	fu51	an35	fin51	10	fu51	an35	fin35
11	fu35	iŋ55	fiŋ55	***11***	fu35	iŋ55	fiŋ35	***11***	fu35	iŋ55	fiŋ55
12	fu51	iŋ35	fiŋ51	***12***	fu51	iŋ35	fiŋ51	***12***	fu51	iŋ35	fiŋ35
13	ku35	i55	ki55	13	ku35	i55	ki35	13	ku35	i55	ki55
14	ku51	i35	ki51	14	ku51	i35	ki51	14	ku51	i35	ki35
15	ku35	an55	kin55	***15***	ku35	an55	kin35	***15***	ku35	an55	kin55
16	ku51	an35	kin51	***16***	ku51	an35	kin51	***16***	ku51	an35	kin35
17	ku35	iŋ55	kiŋ55	17	ku35	iŋ55	kiŋ35	17	ku35	iŋ55	kiŋ55
18	ku51	iŋ35	kiŋ51	18	ku51	iŋ35	kiŋ51	18	ku51	iŋ35	kiŋ35
19	tu35	an55	tin55	***19***	tu35	an55	tin35	***19***	tu35	an55	tin55
20	tu51	an35	tin51	***20***	tu51	an35	tin51	***20***	tu51	an35	tin35

1 All stimuli are transcribed in IPA. 55, 35, 214, 51 represent Mandarin high-level, high-rising, dipping-rising and high-falling tones respectively.

2 Item numbers in bold and italic represent that the items also appear in testing phase.

Training phase—fillers[3]

Language BD				Language LD				Language RD			
	CV	V/VN	CV/CVN		CV	V/VN	CV/CVN		CV	V/VN	CV/CVN
1	wu35	i35	wi35	1	wu214	i214	wi214	1	wu214	i214	wi214
2	wu35	an35	win35	2	wu214	an214	win214	2	wu214	an214	win214
3	wu35	iŋ35	wiŋ35	3	fu214	i214	fi214	3	fu214	i214	fi214
4	fu214	i214	fi214	***4***	fu55	i55	fi55	***4***	fu51	i51	fi51
5	fu214	an214	fin214	***5***	fu214	an214	fin214	***5***	fu214	an214	fin214
6	fu35	an35	fin35	6	fu55	an55	fin55	6	fu51	an51	fin51
7	fu35	iŋ35	fiŋ35	7	fu55	iŋ55	fiŋ55	7	fu51	iŋ51	fiŋ51
8	ku35	i35	ki35	8	ku55	i55	ki55	8	ku51	i51	ki51
9	ku35	an35	kin35	9	ku55	an55	kin55	9	ku51	an51	kin51
10	ku214	iŋ214	kiŋ214	10	ku55	iŋ55	kiŋ55	10	ku51	iŋ51	kiŋ51
11	tu214	an214	tin214	11	tu214	an214	tin214	11	tu214	an214	tin214
12	tu35	an35	tin35	12	tu55	an55	tin55	12	tu51	an51	tin51
13	pa35	in35	pan35	13	ni55	an55	nin55	13	ni51	an51	nin51
14	ta35	iŋ35	taŋ35	***14***	mi55	an55	min55	***14***	mi51	an51	min51
15	pa214	i214	pa214	***15***	pa55	i55	pa55	***15***	pa51	i51	pa51
16	pa214	in214	pan214	***16***	pa214	i214	pa214	***16***	pa214	i214	pa214
17	pa214	iŋ214	paŋ214	17	pa214	in214	pan214	17	pa214	in214	pan214
18	ta35	i35	ta35	***18***	ta214	i214	ta214	***18***	ta214	i214	ta214
19	ta214	i214	ta214	***19***	ta55	in55	tan55	***19***	ta51	in51	tan51
20	ta214	in214	tan214	20	ta214	in214	tan214	20	ta214	in214	tan214
21	fa35	i35	fa35	***21***	fa214	i214	fa214	***21***	fa214	i214	fa214
22	fa214	in214	fan214	22	fa214	in214	fan214	22	fa214	in214	fan214
23	fa214	iŋ214	faŋ214	***23***	fa55	in55	fan55	***23***	fa51	in51	fan51
**24**	fa214	i214	fa214	24	fa55	iŋ55	faŋ55	24	fa51	iŋ51	faŋ51
25	la35	in35	lan35	***25***	la214	i214	la214	***25***	la214	i214	la214
26	la214	i214	la214	26	la55	i55	la55	26	la51	i51	la51
27	la35	iŋ35	laŋ35	27	la214	in214	lan214	27	la214	in214	lan214
28	wa35	iŋ35	waŋ35	28	wa214	i214	wa214	28	wa214	i214	wa214
29	wa214	i214	wa214	29	wa214	in214	wan214	29	wa214	in214	wan214
30	wa214	in214	wan214	***30***	wa55	iŋ55	waŋ55	***30***	wa51	iŋ51	waŋ51

Practice items for the testing phase

Language BD				Language LD				Language RD			
	CV	V/VN	CV/CVN		CV	V/VN	CV/CVN		CV	V/VN	CV/CVN
1	li214	u214	lu214	1	li214	u214	lu214	1	li214	an214	lin214
2	nu35	an35	nin35	2	na55	i55	na55	2	na51	i51	na51

[3] Item numbers in bold and italic represent that the items also appear in testing phase.

Testing phase—unseen critical items

Language BD				Language LD				Language RD			
	CV	V/VN	CV/CVN		CV	V/VN	CV/CVN		CV	V/VN	CV/CVN
1	su35	i55	si55	1	su35	i55	si35	1	su35	i55	si55
2	su51	i35	si51	2	su51	i35	si51	2	su51	i35	si35
3	su35	an55	sin55	3	su35	an55	sin35	3	su35	an55	sin55
4	su51	an35	sin51	4	su51	an35	sin51	4	su51	an35	sin35
5	su35	iŋ55	siŋ55	5	su35	iŋ55	siŋ35	5	su35	iŋ55	siŋ55
6	su51	iŋ35	siŋ51	6	su51	iŋ35	siŋ51	6	su51	iŋ35	siŋ35

Testing phase—unseen fillers

Language BD				Language LD				Language RD			
	CV	V/VN	CV/CVN		CV	V/VN	CV/CVN		CV	V/VN	CV/CVN
1	su35	an35	sin35	1	su55	an55	sin55	1	su51	an51	sin51
2	tu35	iŋ35	tiŋ35	2	lu55	iŋ55	liŋ55	2	lu51	iŋ51	liŋ51
3	ta214	iŋ214	taŋ214	3	ta214	iŋ214	taŋ214	3	ta214	iŋ214	taŋ214

Appendix C. The language background questionnaire for Mandarin speakers

Name: ____

Gender: ____

Age: ____

What Chinese dialects do you speak?

____

____

In comparison to Chinese dialects, is Mandarin Chinese your dominant language?

____

____

When did you start to acquire/learn Mandarin?

____

____

At home, do you speak Mandarin or dialects more often?

____

____

Before going to college, did you speak Mandarin or dialects more often at school?

____

____

Do you speak Mandarin or dialects more often with your friends?

____

____

Appendix D. Self-rating scale for Cantonese speakers' Mandarin proficiency

Name: ______________________________________

Gender: ___________________________________

Age: _______________________________

Native language: ______________________________

Please rate your Mandarin proficiency from Level 0 to 5 according to the following descriptions:

Level 0

- Oral production limited to occasional, isolated words.
- May be able to ask questions or make statements with accuracy only with memorized utterances.

Level 1

- Able to understand basic questions and speech with guides, such as slower speech or repetition, to aid understanding.
- Has a vocabulary only large enough to communicate the most basic of needs.

Level 2

- Able to satisfy routine social demands and limited work requirements.
- Can handle with confidence basic social situations including introductions and casual conversations.
- Can usually handle elementary constructions quite accurately but does not have confident control of the grammar.

Level 3

- Able to speak the language with sufficient structural accuracy and vocabulary to participate effectively in most conversations on practical, social, and professional topics.
- Has a general vocabulary which is broad enough that you rarely has to search for a word.

Level 4

- Able to use the language fluently and accurately on all levels.
- Makes only quite rare and minor errors of pronunciation and grammar.

Level 5

- Has a speaking proficiency equivalent to that of an educated native speaker.

Your level of Mandarin proficiency: ______________________________________
